# Disease-Associated Mutant Tau Prevents Circadian Changes in the Cytoskeleton of Central Pacemaker Neurons

**DOI:** 10.3389/fnins.2020.00232

**Published:** 2020-03-27

**Authors:** Marlène Cassar, Alexander D. Law, Eileen S. Chow, Jadwiga M. Giebultowicz, Doris Kretzschmar

**Affiliations:** ^1^Oregon Institute of Occupational Health Sciences, Oregon Health & Science University, Portland, OR, United States; ^2^Department of Integrative Biology, Oregon State University, Corvallis, OR, United States

**Keywords:** FTDP-17, Tau^V337M^, Tauopathy, sleep disruptions, synaptic homeostasis, PDF neurons, Alzheimer’s disease

## Abstract

A hallmark feature of Alzheimer’s disease (AD) and other Tauopathies, like Frontotemporal Dementia with Parkinsonism linked to chromosome 17 (FTDP-17), is the accumulation of neurofibrillary tangles composed of the microtubule-associated protein Tau. As in AD, symptoms of FTDP-17 include cognitive decline, neuronal degeneration, and disruptions of sleep patterns. However, mechanisms by which Tau may lead to these disturbances in sleep and activity patterns are unknown. To identify such mechanisms, we have generated novel *Drosophila* Tauopathy models by replacing endogenous fly dTau with normal human Tau (hTau) or the FTDP-17 causing hTau^V337M^ mutation. This mutation is localized in one of the microtubule-binding domains of hTau and has a dominant effect. Analyzing heterozygous flies, we found that aged hTau^V337M^ flies show neuronal degeneration and locomotion deficits when compared to wild type or hTau^WT^ flies. Furthermore, hTau^V337M^ flies are hyperactive and they show a fragmented sleep pattern. These changes in the sleep/activity pattern are accompanied by morphological changes in the projection pattern of the central pacemaker neurons. These neurons show daily fluctuations in their connectivity, whereby synapses are increased during the day and reduced during sleep. Synapse formation requires cytoskeletal changes that can be detected by the accumulation of the end-binding protein 1 (EB1) at the site of synapse formation. Whereas, hTau^WT^ flies show the normal day/night changes in EB1 accumulation, hTau^V337M^ flies do not show this fluctuation. This suggests that hTau^V337M^ disrupts sleep patterns by interfering with the cytoskeletal changes that are required for the synaptic homeostasis of central pacemaker neurons.

## Introduction

The microtubule-associated protein Tau is a major component of the neurofibrillary tangles (Grundke-Iqbal et al., 1986) that are a hallmark of Alzheimer’s disease (AD) and a number of other neurodegenerative diseases, together called Tauopathies (Lee et al., 2001; Lee and Leugers, 2012). The relevance of Tau in the pathogenesis of these diseases has been confirmed by the identification of several mutations in Tau that cause Frontotemporal Dementia with Parkinsonism linked to chromosome 17 (FTDP-17). FTDP-17 Tauopathy is a dominant inherited disease and the most prominent symptoms are behavioral and personality changes, cognitive impairment, and motor symptoms (Wszolek et al., 2006). In addition, FTDP-17 patients and other Tauopathy patients show changes in their sleep patterns (McCarter et al., 2016; Holth et al., 2017; Liu et al., 2018). Disturbances in sleep-wake cycles and other circadian rhythms are also very common in Alzheimer patients (van Someren et al., 2007; Reddy and O’Neill, 2010; Kondratova and Kondratov, 2012; Hastings and Goedert, 2013; Musiek et al., 2018; Leng et al., 2019) and recent evidence in AD suggests that sleep abnormalities are not simply a consequence but an intimate and bi-directional component of the pathophysiology. Sleep disruptions impair long-term memory consolidation, thus probably actively contributing to the cognitive decline in AD (Kang et al., 2009; Lim et al., 2014; Roh et al., 2014). A more active role is further supported by findings that sleep disruptions can precede the development of other symptoms of AD, including cognitive decline (Guarnieri and Sorbi, 2015). However, while several studies have suggested a link between the accumulation of plaque-associated β-amyloid and sleep (Brown et al., 2016; Cedernaes et al., 2017; Macedo et al., 2017; Yulug et al., 2017), a role of neuropathic forms of Tau in disrupting sleep has only recently been addressed (Holth et al., 2017). Furthermore, in contrast to amyloid plaques which are characteristic for AD, Tau pathology is found in all Tauopathies and therefore effects of Tau on sleep could account for the sleep disruptions in many Tauopathies, including AD.

Circadian rhythms are controlled by the circadian clock system with the clock in the central pacemaker neurons controlling behavioral rhythms including sleep/wake cycles (Schibler et al., 2003). Daily rhythms are also observed in many other brain functions, such as neuronal plasticity, learning, and memory and generally these rhythms decay during aging (Hastings et al., 2007; Gerstner and Yin, 2010; Reddy and O’Neill, 2010; Smarr et al., 2014). This suggests a neuroprotective function of circadian clocks and, indeed mutations in clock proteins have been shown to cause or aggravate phenotypes in mouse or *Drosophila* models of neurodegenerative diseases (van Someren et al., 1996; Volicer et al., 2001; Harper et al., 2005; Krishnan et al., 2012; Musiek et al., 2013; Musiek, 2015). In contrast, promoting sleep has been shown to reverse the memory deficits observed when expressing the human Amyloid Precursor Protein in *Drosophila* (Dissel et al., 2017). While it has been shown in a variety of models that APP or Aβ expression disrupts sleep, it has only recently been described that a FTPD-17 mouse model shows changes in its sleep patterns. Knock-in mice expressing human Tau (hTau) with the disease-associated P301L or R406W mutation in the forebrain reveal decreased non-REM sleep and increased wakefulness (Koss et al., 2016; Holth et al., 2017). To study the emerging connection between mutant Tau and sleep, we used the *Drosophila* model to create knock-in flies that either express wild type human Tau (hTau^WT^) or FTDP-17-causing mutant hTau^V337M^ instead of *Drosophila* Tau (dTau). In addition to a disruption of their sleep pattern, hTau^V337M^ flies showed changes in the axonal pattern of central pacemaker neurons and in their synaptic connection, suggesting that cytoskeletal alterations caused by this mutation prevent the synaptic homeostasis in sleep-regulating neurons.

## Materials and Methods

### Drosophila Stocks

The knock-in lines were created by cloning a cDNA encoding the hTau 1N4R isoform (kindly provided by B. Kraemer, University of Washington) into the pHD-DsRed-attp vector together with 1.8 kb of the sequence upstream of the dTau coding region and 1.2 kb of the sequence downstream of dTau. hTau was inserted into the dTau coding region using the CRISPR/Cas9 genomic editing system (Supplementary Figure S1A) and guide RNAs (Supplementary Figure S1B) cloned into the pBTv-U6.2 vector using the BestGene injection service. UAS-GFP-tubulin was kindly provided by D. Applewhite (Reed College), UAS-EB1-GFP by M. Rolls (PennState University), and UAS-mCD4-GFP by M. Logan (OHSU). The *Pdf*-GAL4 lines are described in Renn et al. (1999). The dTau knock-out line was provided by the Bloomington Drosophila Stock Center. Flies were maintained on standard fly food under a 12:12h light:dark cycle at 26°C.

### Fast Phototaxis

Fast phototaxis assays were conducted in the dark using the countercurrent apparatus described by Benzer (1967) and a single light source. A detailed description of the experimental conditions can be found in Strauss and Heisenberg (1993). Flies were collected every day and aged to the given age with fresh food vials provided every 4–5 days. Flies were then tested in groups of 10–15 flies. Five consecutive tests were performed in each experiment with a time allowance of 6 s to make a transition toward the light and into the next vial and a value determined for each fly based on which of the six vials it reached. Statistical analysis was done using GraphPad Prism and one-way ANOVA with Dunnett’s post-tests.

### Tissue Sections and Vacuole Measurements

Flies were obtained and aged as described for the phototaxis experiments. Paraffin sections for light microscopy were prepared and analyzed for vacuole formation as described in Botella et al. (2003) and Sunderhaus and Kretzschmar (2016). Briefly, whole flies were fixed in Carnoy’s solution and dehydrated in an ethanol series followed by incubation in methyl benzoate before embedding in paraffin. Sections were cut at 7 μm and analyzed with a Zeiss Axioscope 2 microscope using the auto-fluorescence caused by the dispersed eye pigment. Semi-thin and ultrathin Epon plastic sections were prepared as described in Kretzschmar et al. (1997). Semi-thin sections were cut at 1 μm and stained with toluidine blue. Ultra-thin sections were cut at 50 nm and electron microscopic images taken with a FEI Tecnai G2 microscope. To quantify the vacuolization, we photographed the paraffin sections that contained the antennal lobes and the most prominent vacuolization in each fly head without knowing the genotype. For a double-blind analysis, pictures were numbered and the number of vacuoles in the antennal lobes and AMMC counted before the genotype was revealed. Statistical analysis was done using GraphPad Prism and one-way ANOVA with Dunnett’s post-tests to compare to CS and Student’s *t*-test to compare the two knock-in lines.

### Immunohistochemistry

For whole-mounts, brains were dissected in ice-cold PBS and transferred to 4% PFA in PBS. They were then fixed for 30 min to 1 h at room temperature (RT) and washed four times with PBS/0.5% Triton (PBS-T) for 10 min each before blocking with 5% normal goat serum in PBS overnight at 4^*o*^C. To detect GFP, EB1-GFP, and GFP-tubulin, anti-GFP (Thermo Fisher Scientific A-11122) was used at 1:250 overnight at 4°C. Brains were then washed three times in PBS, 20 min each at RT and the secondary antibody applied (anti-rabbit-Cy2, Jackson ImmunoResearch) at 1:250 for 2 h at RT. Anti-Tau13 (Abcam ab19030) was used at 1:100 and detected with anti-mouse-Cy3 (VectorLabs) at 1:1000 or the anti-mouse Vecta Fluor Antibody kit (VectorLabs, DK-2488) following the instruction manual. Brains were washed three times for 20 min with PBS and mounted in Glycergel for confocal imaging using an Olympus FluoView 300 laser scanning confocal head mounted on an Olympus BX51 microscope.

### Western Blots

To detect hTau, we modified a protocol from Feuillette et al. (2010). Thirty-five adult fly heads were dissected on an ice-cold plate, homogenized in 100 μl of RIPA lysis buffer [150 mM NaCl, 1% DOC, 1% SDS, 50 mM Tris, 5 mM EDTA, 5 mM EGTA, 1% triton X-100, and protease inhibitors (Cell Signaling Technology 5872S)], and immediately centrifuged at 10,000 × *g* for 10 min at 4°C. The supernatant was discarded and the pellet was homogenized in 100 μl of 70% formic acid and then incubated for 30 min at 37°C. Samples were centrifuged again at 10,000 × *g* for 10 min at 4°C. The supernatant was transferred to a fresh tube and the formic acid was evaporated by vacuum centrifugation. The resulting pellet was resuspended in 30 μl of 1.25 × LDS sample buffer (Thermo Fisher Scientific B0008), supplemented with 50 mM tris(2-carboxyethyl)phosphine (TCEP) as a reducing agent, and immediately denatured at 95°C for 5 min. Samples were stored at -20°C overnight, denatured again at 95°C for 5 min, and loaded onto 8% bis-tris gels (Thermo Fisher Scientific NW00082). After transfer, PVDF membranes (GE Healthcare 106000230) were blocked with 1× casein blocking buffer (Sigma C7594). Primary antisera/antibodies were used at the following dilution: mouse anti-tau 5 (1:200; Invitrogen MA5-12808) and mouse anti-GAPDH G-9 (1:1000; Santa Cruz sc-365062) incubated over night at 4°C. To detect hTau and GAPDH, we used a biotinylated secondary antibody (Vector Labs BA-2000) and Streptavidin-conjugated alkaline phosphatase (Vector Labs AK-6000) following the manufacturer protocol with the exception that all washing steps were carried out with 1× TBST. Enhanced chemiluminescent substrate (Vector Labs SK-6605) was used to visualize bands.

### Locomotor Activity and Sleep Analysis

In two independent experiments, at least 22 adult males from each specified age and genotype were held individually in glass tubes containing diet in one end, and a piece of yarn plugging the other end. Tubes were placed in Drosophila Activity Monitors (DAM) models DAM2 or DAM5 (Trikinitecs, Waltham, MA, United States) to measure locomotor activity (the monitors are different sizes but hold the same tubes and take the same readings). Activity counts were taken once every minute for three days of light/dark (12h:12h LD), followed by at least seven days of constant darkness (DD). Activity experiments were performed at 25 ± 1°C and ∼1000–1200 l× during light phase. Analysis of activity counts, rhythmicity as measured by fast Fourier transform (FFT), and sleep was performed with ClockLab 6 (Actimetrics, Wilmette, IL, United States). Sleep bouts were defined as a 5-min interval in which no activity was detected. Graphs and statistical tests of data were done in GraphPad Prism 6 (San Diego, CA, United States). The indicated ages were their age at the beginning of the activity recording.

## Results and Discussion

### hTau^V337M^ Knock-in Flies Show Degeneration and Locomotion Deficits

**FIGURE 1:**
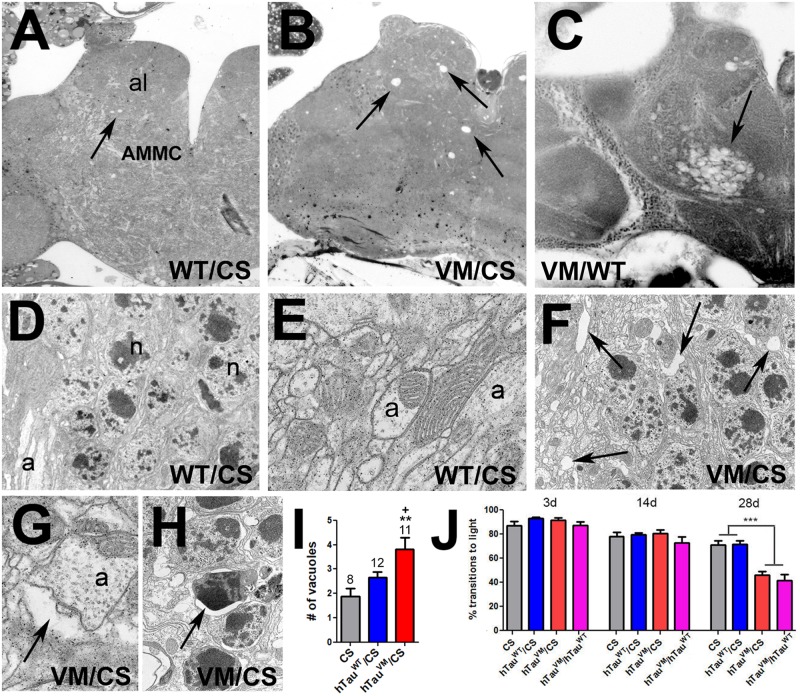
**(A)** A 60-day-old hTau^WT^/CS fly shows a few small vacuoles in the antennal lobes and AMMC (arrow). More and larger vacuoles are found in age-matched hTau^V337M^/CS **(B)** and hTau^V337M^/hTau^WT^ **(C)**. **(D,E)** EM images from hTau^WT^/CS showing intact neuronal cell bodies (**D**, n = nucleus) and neurites (**E**, a = axon). **(F)** In hTau^V337M^/CS, gaps appear between cell bodies (arrows) and around neurites (**G**, arrow). **(H)** Dark, shrunken nuclei, indicative of neuronal death, are also detectable in hTau^V337M^/CS (arrow). **(I)** Number of vacuoles in the al and AMMC. Number of flies used indicated. **(J)** Heterozygous hTau^V337M^/CS or hTau^V337M^/hTau^WT^ flies show reduced locomotion when 28-day-old. At least 40 flies were tested for each genotype and age. Error bars indicate SEMs. One-way ANOVA with Dunnett’s Post Test was used to compare to CS (*) and a Student’s *t*-test to compare hTau^V337M^/CS to hTau^WT^/CS (+). ^+^*p* < 0.05, **p < 0.01, ***p < 0.001.

To investigate mechanisms by which mutations in Tau lead to pathology, we generated two knock-in lines in which dTau was replaced by the coding region of either normal hTau (hTau^WT^) or FTDP-17-associated hTau^V337M^. We confirmed the removal of dTau and correct insertion of the hTau sequence into the endogenous dTau gene in both knock-in lines by PCR (data not shown) and Western blots (Supplementary Figure S2A). We also confirmed that hTau is expressed in the CNS by immunohistochemistry (Supplementary Figures S2B,C). The hTau^V337M^ mutation was first identified in a Seattle family with an autosomal-dominant pattern of inheritance (Poorkaj et al., 1998) and as the name implies, FTDP-17 affects the frontal and temporal lobes, leading to neuronal loss and brain atrophy in the patients. We therefore tested whether we could detect degenerative phenotypes in aged heterozygous flies expressing hTau^V337M^. Whereas we did not detect overt signs of degeneration in toluidine-stained tissue sections from heterozygous 30-day-old hTau^V337M^/CS (Canton S wild type, Figure 1A) flies, by 60-day spongiform lesions had formed, primarily in the antennal lobes (al) and the antennal mechanosensory and motor center (AMMC) of hTau^V337M^/CS flies (arrows, Figure 1B). Comparing the number of vacuoles in the antennal lobes and AMMC confirmed a significant increase in 60-day-old hTau^V337M^/CS compared to age-matched CS and hTau^WT^/CS (Figure 1I). We also analyzed 60-day-old hTau^V337M^/hTau^WT^ flies and again found more and larger vacuoles in these areas but in addition some of these flies showed a spongiform appearance over a wide area in the AMMC (arrow, Figure 1C) which made it difficult to identify single vacuoles. Analyzing EM sections from these flies, we found small vacuoles and empty spaces between neuronal cell bodies (arrows, Figure 1F) and neurites (arrow, Figure 1G) in 60-day-old Tau^V337M^/CS in addition to shrinking and dying cells (arrow, Figure 1H). In contrast, age-matched hTau^WT^/CS did not show these phenotypes (Figures 1D,E). Besides neuronal loss, prominent symptoms in FTDP-17 patients are behavioral and personality changes, as well as mobility impairments (Wszolek et al., 2006). To determine whether our model also reveals locomotion deficits, we performed fast phototaxis assays in which the flies are given 6 s to run toward a light source. Whereas no difference was detected in 3- and 14-day-old flies, hTau^V337M^/CS performed significantly worse when aged to 4 weeks compared to hTau^WT^/CS or CS (Figure 1J). A similar reduction in performance was also detected in 28-day-old heterozygous hTau^V337M^/hTau^WT^ flies. This shows that hTau^V337M^ has dominant effects as in human patients and that it does induce locomotion deficits and degeneration in aged flies. The degeneration was most prominent in the antennal lobes and especially the AMMC, which has been show to play a role in a variety of behaviors, including social behaviors connected with courtship and locomotor responses triggered by air flow (Patella and Wilson, 2018). Whereas the behavioral deficits were already seen in mid-aged flies, the degeneration only became detectable in old flies, suggesting that changes in neuronal function may cause the locomotion deficits rather than neuronal degeneration.

### hTau^V337M^ Disrupts Sleep/Activity Patterns but Not Rhythmicity

**FIGURE 2:**
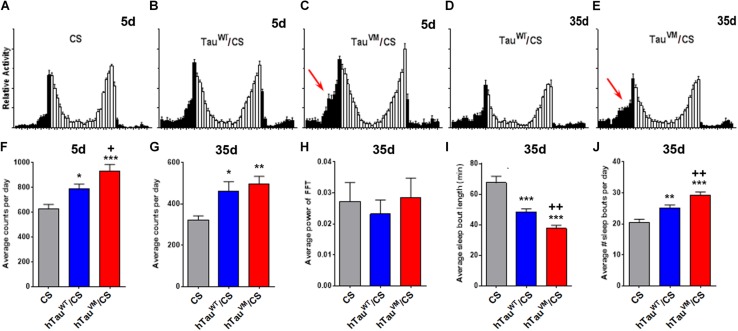
**(A–E)** Activity patterns show increased activity toward the end of the night (black bars) in 5- and 35-day-old hTau^V337M^/CS (arrows in **C,E**). **(F)** Counting the activity bouts per day shows an increase in 5-day-old hTau^WT^/CS and hTau^V337M^/CS compared to CS (asterisks) but hTau^V337M^/CS is also significantly more active than hTau^WT^/CS (plus sign). **(G)** At 35-day hTau^WT^/CS and hTau^V337M^/CS are more active than CS but no significant difference is found between hTau^WT^/CS and hTau^V337M^/CS. **(H)** Behavioral rhythmicity is not altered in hTau^WT^/CS or hTau^V337M^/CS. **(I,J)** Sleep is fragmented in 35-day-old hTau^WT^/CS and hTau^V337M^/CS compared to CS but hTau^V337M^/CS also shows significantly shorter and more sleep bouts than hTau^WT^/CS. * one-way ANOVA with Dunnett’s Post Test comparing the hTau/CS lines to CS. ^+^Student’s *t*-test comparing hTau^WT^/CS and hTau^V337M^/CS. *n* > 22. *p < 0.05, **p < 0.01, ***p < 0.001, ^+^*p* < 0.05, ^++^*p* < 0.01.

As mentioned above, increasing evidence link Tauopathies with sleep and other circadian disruptions (Musiek and Holtzman, 2016). We therefore analyzed activity and sleep patterns in the knock-in flies. Compared to CS, both 5-day-old hTau^WT^/CS and hTau^V337M^/CS flies kept in 12:12 light/dark (LD) cycles showed an increase in activity. However, hTau^V337M^/CS was also significantly more active than hTau^WT^/CS (Figures 2A–C,F), especially during the late night (arrow in Figure 2C). When 35-day-old, all the flies were less active but again both knock-in flies were hyperactive compared to CS. At this age hTau^V337M^/CS was not significantly different from hTau^WT^/CS (Figures 2D,E,G). As shown in Figure 1J, hTau^V337M^/CS flies showed a significant decline in the fast phototaxis assays at this age and we therefore assume that the reduced locomotion at this age is preventing hyperactivity in 35-day-old hTau^V337M^/CS. Maintaining the flies in constant darkness showed that hTau^V337M^ did not affect circadian behavioral rhythmicity when 5-day-old (data not shown) or 35-day-old (Figure 2H). The free-running period was 24.1 h for hTau^V337M^/CS and 23.9 h for hTau^WT^/CS. The % rhythmic was 84.4% for hTau^V337M^/CS and 82.9% for hTau^WT^/CS. Analyzing the sleep pattern of these flies by measuring sleep bout length and number of sleep bouts, we did not find any differences in 5-day-old flies (data not shown). However, 35-day-old knock-in flies showed a shorter sleep bout length with hTau^V337M^/CS being significantly worse than hTau^WT^/CS (Figure 2I). Counting the number of sleep bouts we found an increase in the average number per day and again hTau^V337M^/CS was more affected than hTau^WT^/CS (Figure 2J). Plotting the time of sleep over the day, 5-day-old flies did not show a change in daytime naps but sleep was reduced during the end of the night (more prominently in hTau^V337M^/CS, Supplementary Figure S3A), consistent with the increased activity observed during that time. This was also detectable in 35-day-old hTau^V337M^/CS flies which in addition showed a decrease in daytime naps in the morning while hTau^WT^/CS flies did not (Supplementary Figure S3B). Together, these results show that both hTau expressing lines do affect the sleep pattern compared to CS. That hTau^WT^/CS also showed changes in the sleep pattern may be due to it not being completely able to substitute for *Drosophila* dTau. This is supported by the recent finding that the loss of dTau reduces sleep and increases activity (Arnes et al., 2019). Furthermore, we found that also haploinsufficiency by using a dTau knock-out-line (Burnouf et al., 2016), impaired the sleep pattern (Supplementary Figure S3C) and induced hyperactivity when tested at 5-day (Supplementary Figure S3D). However, hTau^V337M^ was significantly worse than hTau^WT^, showing that the mutation does impair the function of Tau in regulating sleep.

### Axonal Terminals of Central Pacemaker Neurons Are Altered in Tau^V337M^/CS Flies

**FIGURE 3:**
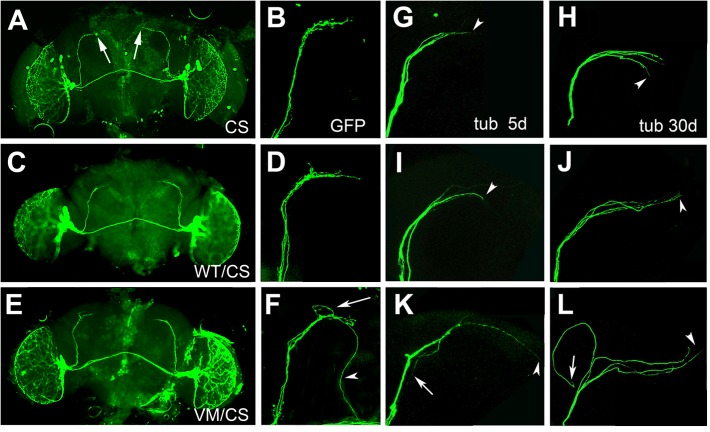
**(A–F)** mCD4-GFP expressed via *Pdf*-GAL4 in 30-day-old flies. CS **(A,B)** and heterozygous hTau^WT^/CS **(C,D)** show the normal axonal pattern. **(E,F)** In hTau^V337M^/CS the branching in the target area appears more spread out (arrow in **F**) and some axons are elongated (arrowhead). **(G–L)** GFP-tubulin expression via *Pdf*-GAL4. Termination area of PDF neurons in 5-day **(G)** and 30-day-old CS **(H)**. hTau^WT^/CS also show the regular pattern when 5-day-old **(I)** but occasionally extended axons are detected when 30-day-old (**J**, arrowhead). **(K)** hTau^V337M^/CS show elongated (**K**, arrowhead) and misrouted (arrow) axons already when 5-day-old. **(L)** In a 30-day-old hTau^V337M^/CS fly, three of the axons extend beyond the target area (arrowhead), while another one turned into the opposite direction (arrow).

As described above, hTau^V337M^/CS flies show increased wakefulness during the late evening and in the morning. Morning activity is largely determined by the small ventrolateral neurons (sLNvs) that express the Pigment dispersing factor (PDF) (Tataroglu and Emery, 2014; Guo et al., 2018). The sLNvs form a small group of neurons, with four neurons in each hemisphere, that send their axons in a well described pattern to the dorsomedial protocerebrum (arrows, Figure 3A) (Helfrich-Forster et al., 2007; Tomioka and Matsumoto, 2009). We therefore tested whether the changes in the activity/rest pattern in hTau^V337M^/CS could be due to effects on the PDF neurons. Expressing mCD4-GFP with *Pdf*-GAL4 revealed the normal arborization pattern in 30-day-old CS and in hTau^WT^/CS (Figures 3A–D). However, 30-day-old hTau^V337M^/CS flies showed an increase in branching in the termination field (Figures 3E,F, arrow) and some axons that extended beyond their normal target area (arrowhead). To determine whether this correlates with alteration in the cytoskeleton, we expressed GFP-tubulin via *Pdf*-GAL4. We also analyzed 5- and 30-day-old flies to address whether this phenotype is progressive. As expected, 5- and 30-day-old CS flies showed the normal projection pattern (Figures 3G,H) and so did 5-day-old hTau^WT^/CS flies (Figure 3I). When 30-day-old, hTau^WT^/CS occasionally showed elongated projections (arrowhead, Figure 3J). In contrast, in hTau^V337M^/CS some axons extended beyond their target area already when 5-day-old (arrowhead, Figure 3K) while others turned back toward the cell bodies (arrow, Figure 3K). This phenotype became more prominent at 30-day, with most axons extending beyond their termination field (arrowhead, Figure 3L) or turning into different directions (arrow). The phenotype appeared stronger when expressing GFP-tubulin compared to mCD4-GFP, suggesting that the expression of additional tubulin promotes this phenotype. To quantify these changes, we grouped the terminals into three categories; normal, increased branching, and elongated and found that about 90% of the terminals in 30-day-old hTau^V337M^/CS fell into the latter two groups (Supplementary Figure S4). Because *in vitro studies* showed that the V337M mutation impaired its function in stabilizing microtubules (Hasegawa et al., 1998; Hong et al., 1998), we analyzed microtubules size in EM sections. While we did find an increase in the mean cross-sectional area of microtubules in 60-day-old hTau^V337M^/hTau^V337M^ flies compared to CS or hTau^WT^/hTau^WT^, this was only the case when homozygous (Supplementary Figure S5A) but not when heterozygous (Supplementary Figure S5B). Although this confirms an effect of the mutation on the microtubules-stabilizing function *in vivo*, this does not seem to play a role in heterozygotes and therefore the changes in the axonal morphology of PDF neurons in hTau^V337M^/CS are not caused by effects on microtubule formation or stability.

### Tau^V337M^ Interferes With Circadian Changes in the Morphology of PDF Neurons

**FIGURE 4:**
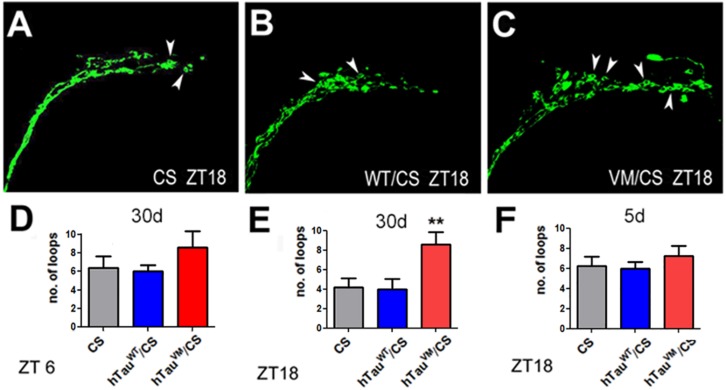
EB1-GFP loops (arrowheads) in the axonal terminals of PDF neurons of 30-day-old CS **(A)**, hTau^WT^/CS **(B)**, and hTau^V337M^/CS **(C)**. Preparations were obtained at ZT 18. Counting the number of loops in 30-day-old flies showed no difference at ZT6 **(D)** but at ZT18 the number of loops was increased in hTau^V337M^/CS compared to controls **(E)**. When 5-day-old, hTau^V337M^/CS did not show an increase in EB1-positive loops at ZT18 **(F)**. 10 flies were analyzed for each bar. One-way ANOVA with a Dunnett’s Multiple Comparison’s test used. *p < 0.05, **p < 0.01.

The PDF neurons regulate sleep as part of a network (Guo et al., 2018) and the changes in the termination pattern of the PDF neurons could therefore interfere with their connectivity with other neurons. PDF neurons show daily fluctuations in their connectivity, whereby synapses are potentiated during the day and altered or downscaled during sleep, a process generally referred to as “synaptic homeostasis” (Tononi and Cirelli, 2003, 2014). Remodeling of synapses requires cytoskeletal changes and it has been shown that the end-binding protein 1 (EB1), needed for microtubules to grow at their plus ends (Akhmanova and Steinmetz, 2010), accumulates in loops around forming synapses (Wang et al., 2007; Stone et al., 2008; Conde and Caceres, 2009). To determine whether hTau^V337M^ interferes with the cytoskeletal changes required for synaptic homeostasis of the PDF neurons, we expressed EB1-GFP via *Pdf*-GAL4 in PDF neurons and counted the number of loops in the terminals of the sLNvs (arrowheads, Figures 4A–C). Counting loops during the day (ZT5-6) in 30-day-old flies did not reveal a significant difference, although the number was slightly higher in hTau^V337M^/CS (Figure 4D). However, counting during the night (ZT17-18) when the PDF neurons normally show less complexity and a reduced number of synapses (Fernández et al., 2008; Gorostiza et al., 2014), a significant increase in loops was detected in hTau^V337M^/CS compared to controls (Figure 4E). As expected, the number of loops was decreased in the controls; from 6.4 to 4.2 in CS and from 6.0 to 4.0 in hTau^WT^/CS however, in hTau^V337M^/CS the mean number of loops was the same during the night as during the day (8.6). Lastly, to determine whether this phenotype is affected by age, we counted loops in 5-day-old flies at ZT17-18. Because we did not detect a significant difference in hTau^V337M^/CS flies compared to the controls (Figure 4F), the effect on synaptic homeostasis appears to increase with age, correlating with the progressively worsening sleep fragmentation.

Together, our findings suggest that the disease-associated hTau^V337M^ has a reduced ability to support the cytoskeletal changes that are required for the day/night synaptic adaptations of PDF neurons. Over time, this results in a failure to downscale synapses during the night, thereby affecting the connectivity within the sleep circuit. This failure of synaptic homeostasis and appropriate changes in connectivity would then cause the sleep disruptions and hyperactivity. In addition to the sleep disruptions, we also detected locomotion deficits. While we do not think that this phenotype is due to the changes in PDF neurons it may also be caused by altered synaptic contacts of neurons that regulate locomotion. Similarly it remains to be determined whether cytoskeletal changes eventually lead to the degeneration.

## Data Availability Statement

The raw data supporting the conclusions of this article will be made available by the authors, without undue reservation, to any qualified researcher.

## Author Contributions

MC generated the knock-in lines and performed the fast phototaxis and immunohistochemical experiments as well as Western blots. AL also performed Western blots, fast phototaxis experiments, and tissue sections. EC performed the activity and sleep experiments. MC, JG, and DK designed and analyzed the experiments. DK wrote the manuscript.

## Conflict of Interest

The authors declare that the research was conducted in the absence of any commercial or financial relationships that could be construed as a potential conflict of interest.

## Supplementary Material

FIGURE S1 **(A)** Schematic of the knock-in approach. **(B)** Sequence of the used guide RNAs (highlighted in yellow). The 5’ guide is localized in exon 1 and the 3’ guide in exon 5 relating to isoform H (which contains the first and last exon in the dTau coding region).

FIGURE S2 **(A)** Whereas no dTau (upper panel) is detectable in the homozygous knock-in lines, they do express hTau (lower panel). α-GAPDH was used as loading control. Immunohistochemistry showing hTau in the CNS of hTau^WT^ flies **(C)** but not in wild type CS **(B)**. Flies were 1–3-day-old.

FIGURE S3 **(A)** Nighttime sleep is shortened in 5-day-old hTau^V337M^/CS compared to hTau^WT^/CS and CS (arrow). **(B)** 35-day-old hTau^V337M^/CS also show shortened nighttime sleep (arrow) and less rest periods at the beginning of the day (arrowhead). **(C)** Like hTau^WT^/CS and hTau^V337M^/CS, 35-day-old dTau^*KO*^/CS show less naps during the end of the day (arrowhead). **(D)** 5-day-old dTau^*KO*^/CS flies are more active than CS. At least 30 flies analyzed. Mean and SEM indicated. Statistics done with Student’s *t*-test. ^∗∗^*p*<0.01.

FIGURE S4 Average percentage of GFP-tubulin labeled PDF terminals with normal (lower part), more spread out (middle) or elongated axons (upper) in CS, hTau^WT^/CS, and hTau^V337M^/CS. Ten 30-day-old flies analyzed for each genotype.

FIGURE S5 **(A)** Microtubule size, measured as cross sectional area, is increased in 60-day-old homozygous hTau^V337M^ compared to hTau^WT^ and CS. **(B)** Microtubules size is not different from controls in heterozygous hTau^V337M^/CS. Between 52 and 123 microtubules were analyzed from at least 50 neurites from 3 flies for each genotype. Horizontal lines are medians; boxes are 25 and 75% quartiles; whiskers are 10 and 90% quantiles. Statistics done with one-way ANOVA and a Dunnett’s Multiple Comparison’s to compare means. ^∗^*p*<0.05.
