# Supplementary figures and images for: Disease-Associated Mutant Tau Prevents Circadian Changes in the Cytoskeleton of Central Pacemaker Neurons
Source: Front Neurosci. 2020 Mar 27;14:232. doi: 10.3389/fnins.2020.00232 (PMC7118733; doi:10.3389/fnins.2020.00232)

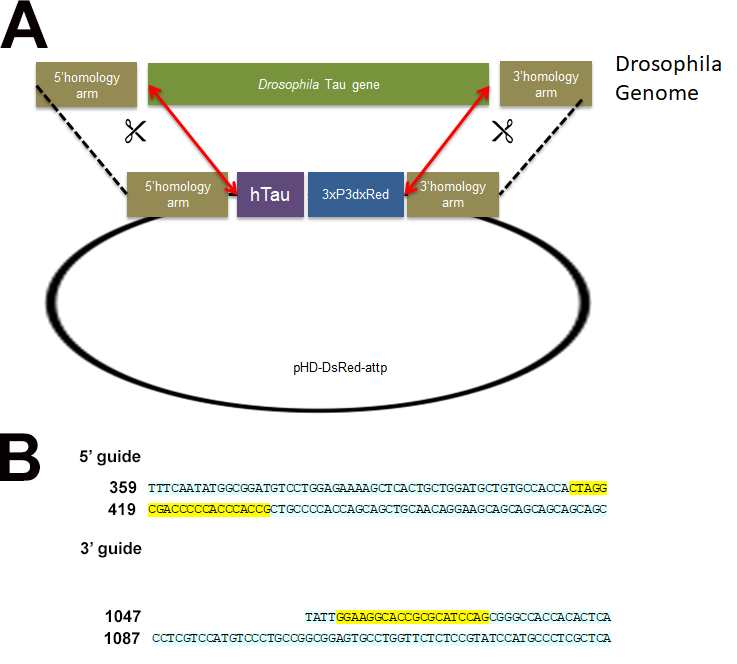

Supplement: FIGURE S1 — (A) Schematic of the knock-in approach. (B) Sequence of the used guide RNAs (highlighted in yellow). The 5’ guide is localized in exon 1 and the 3’ guide in exon 5 relating to isoform H (which contains the first and last exon in the dTau coding region). [file Image_1.TIF]

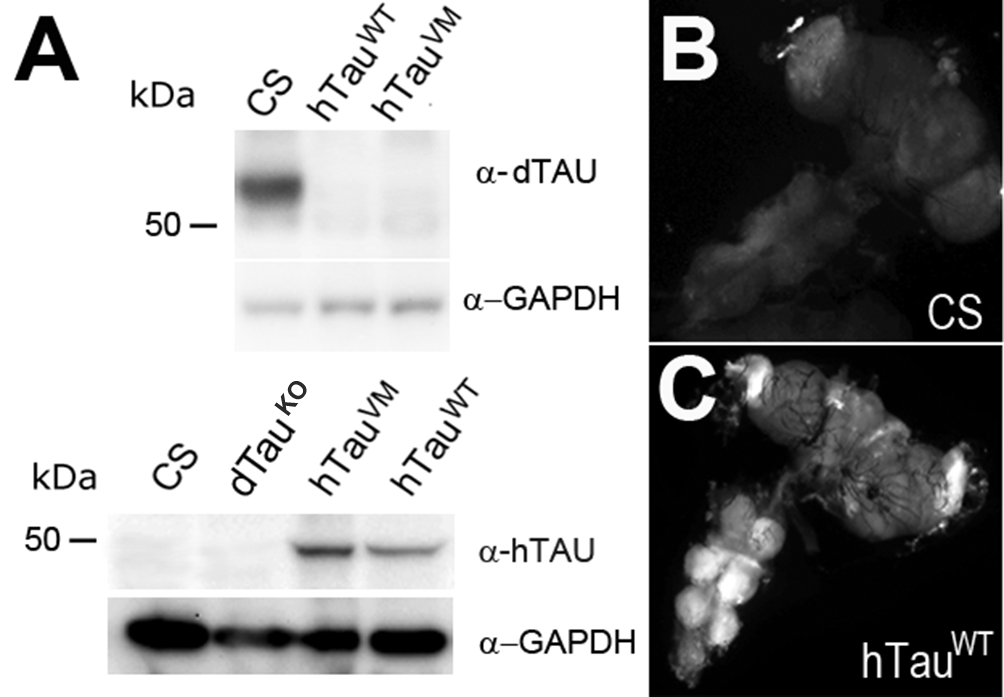

Supplement: FIGURE S2 — (A) Whereas no dTau (upper panel) is detectable in the homozygous knock-in lines, they do express hTau (lower panel). α-GAPDH was used as loading control. Immunohistochemistry showing hTau in the CNS of hTauWT flies (C) but not in wild type CS (B). Flies were 1–3-day-old. [file Image_2.TIF]

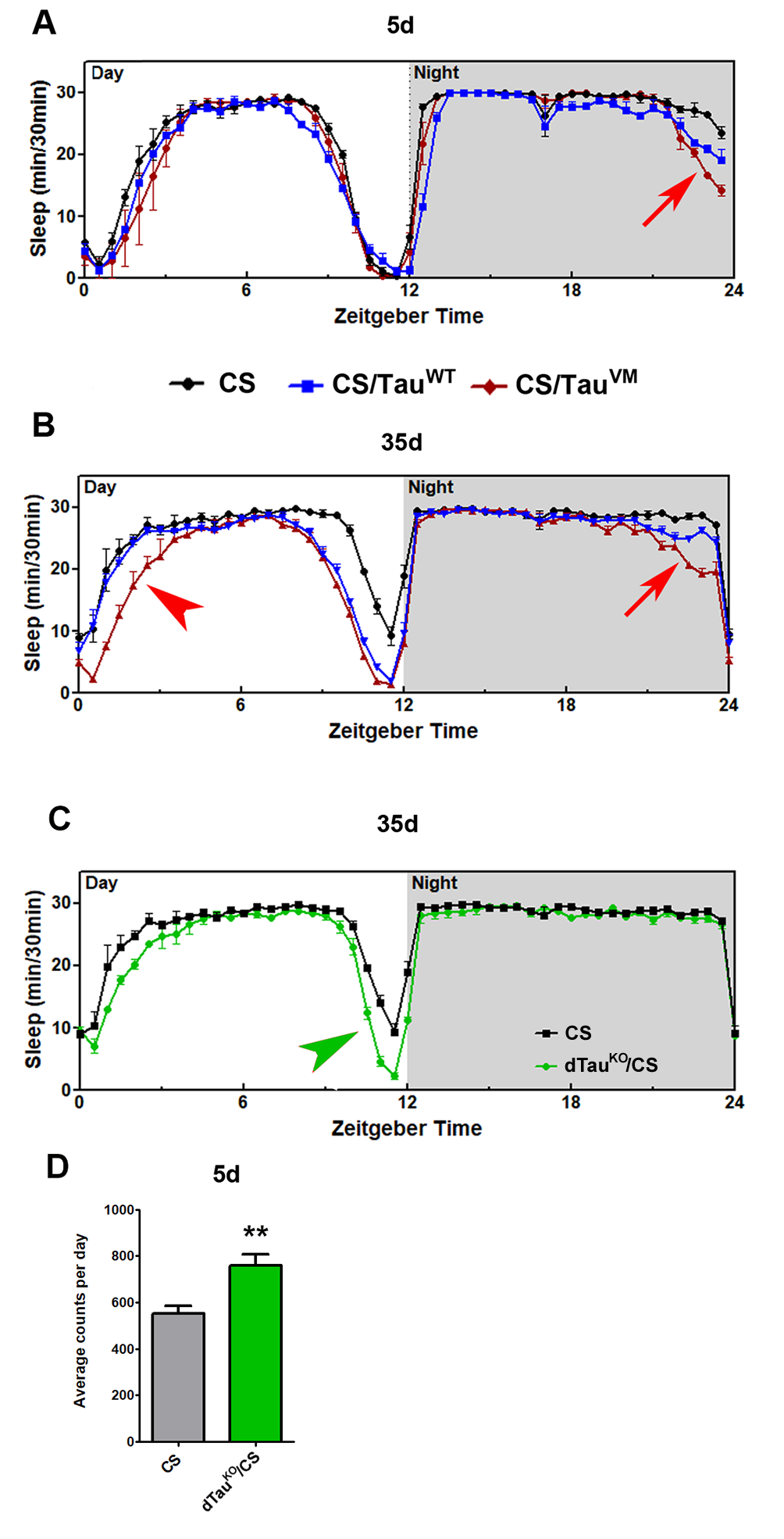

Supplement: FIGURE S3 — (A) Nighttime sleep is shortened in 5-day-old hTauV337M/CS compared to hTauWT/CS and CS (arrow). (B) 35-day-old hTauV337M/CS also show shortened nighttime sleep (arrow) and less rest periods at the beginning of the day (arrowhead). (C) Like hTauWT/CS and hTauV337M/CS, 35-day-old dTauKO/CS show less naps during the end of the day (arrowhead). (D) 5-day-old dTauKO/CS flies are more active than CS. At least 30 flies analyzed. Mean and SEM indicated. Statistics done with Student’s t-test. ∗∗p<0.01. [file Image_3.TIF]

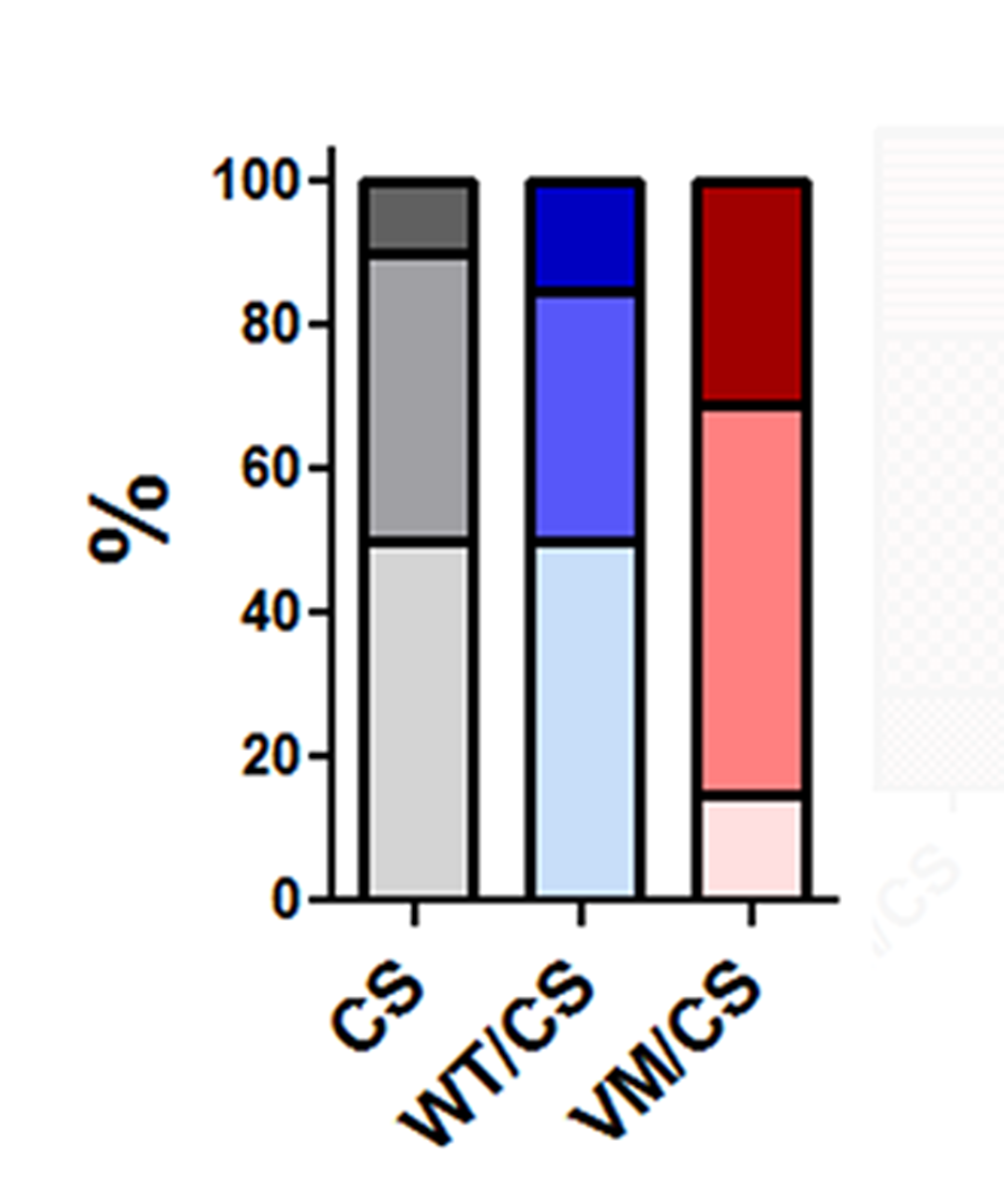

Supplement: FIGURE S4 — Average percentage of GFP-tubulin labeled PDF terminals with normal (lower part), more spread out (middle) or elongated axons (upper) in CS, hTauWT/CS, and hTauV337M/CS. Ten 30-day-old flies analyzed for each genotype. [file Image_4.TIF]

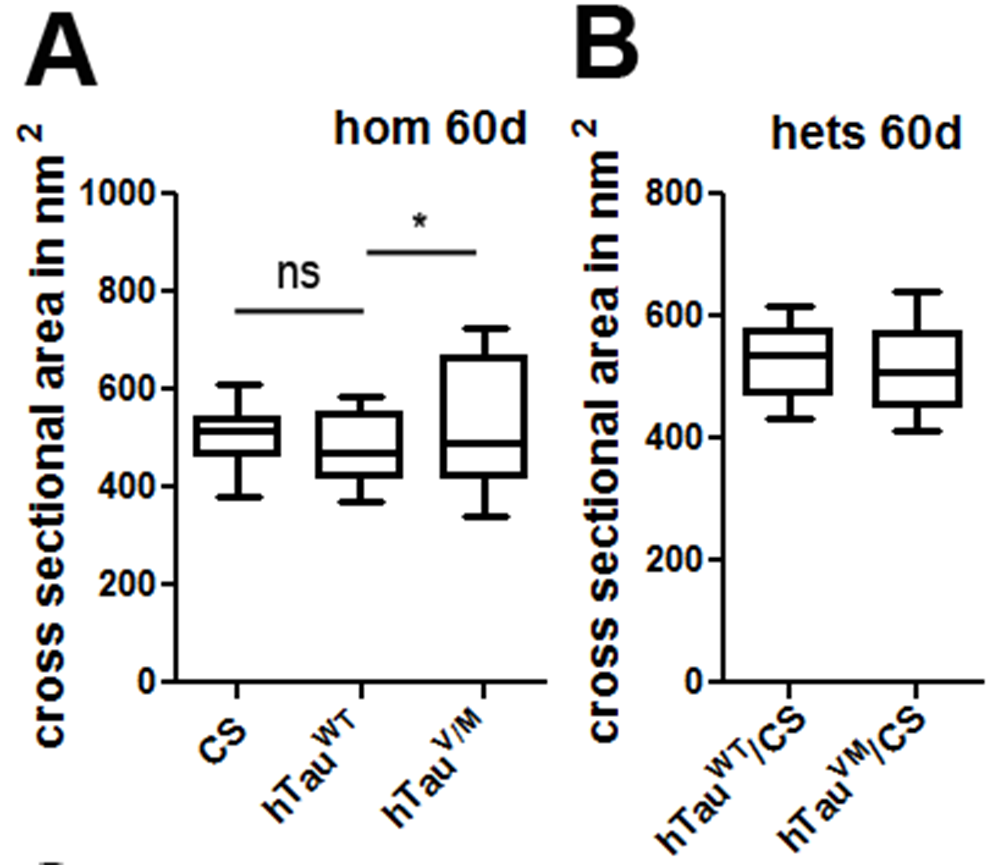

Supplement: FIGURE S5 — (A) Microtubule size, measured as cross sectional area, is increased in 60-day-old homozygous hTauV337M compared to hTauWT and CS. (B) Microtubules size is not different from controls in heterozygous hTauV337M/CS. Between 52 and 123 microtubules were analyzed from at least 50 neurites from 3 flies for each genotype. Horizontal lines are medians; boxes are 25 and 75% quartiles; whiskers are 10 and 90% quantiles. Statistics done with one-way ANOVA and a Dunnett’s Multiple Comparison’s to compare means. ∗p<0.05. [file Image_5.TIF]
